# Discovery of Potential Candidate Genes for Coat Colour in Wuzhishan Pigs by Integrating SNPs and mRNA Expression Analysis

**DOI:** 10.3390/ani14233493

**Published:** 2024-12-03

**Authors:** Qiao Xu, Yabiao Luo, Zhe Chao, Jibin Zhang, Xiaolei Liu, Danqin Tu, Qin Guo, Ruiping Sun, Feng Wang, Meiying Fang

**Affiliations:** 1 Jiangxi Provincial Key Laboratory of Poultry Genetic Improvement, Institute of Biological Technology, Nanchang Normal University, Nanchang 330032, China; xuqiao987@163.com (Q.X.); tudanqin@163.com (D.T.); g13407961498@163.com (Q.G.); 2 National Engineering Laboratory for Animal Breeding, MOA Key Laboratory of Animal Genetics and Breeding, Beijing Key Laboratory for Animal Genetic Improvement, State Key Laboratory of Animal Biotech Breeding, Frontiers Science Center for Molecular Design Breeding, Department of Animal Genetics and Breeding, College of Animal Science and Technology, China Agricultural University, Beijing 100193, China; yabiaoluo@cau.edu.cn; 3 Institute of Animal Sciences and Veterinary, Hainan Academy of Agricultural Sciences, Haikou 571100, China; chaozhe@hnaas.org.cn (Z.C.); ruiping937@126.com (R.S.); wfeng73cn@sina.com (F.W.); 4 Department of Cell and Molecular Biology, Beckman Research Institute of the City of Hope, Duarte, CA 91010, USA; jibinzhang12@gmail.com; 5 Key Laboratory of Agricultural Animal Genetics, Breeding, and Reproduction of Ministry of Education & Key Laboratory of Swine Genetics and Breeding of Ministry of Agriculture, Huazhong Agricultural University, Wuhan 430070, China; xiaoleiliu@mail.hzau.edu.cn; 6 Sanya Institute of China Agricultural University, Sanya 572025, China

**Keywords:** Wuzhishan pig, coat colour, GWAS, *RAPGEF2*, *PDGFRA*, *KIT*

## Abstract

Despite identifying genes regulating the coat colour in Western pig breeds, the genetic basis of the coat colour in Chinese indigenous pigs is still not understood due to the diversity of indigenous breeds and their genetic differences from exotic pigs. In this study, 215 Wuzhishan pigs with three coat colour patterns (white, black, and black-back/white-belly) were used to conduct a genome-wide association analysis. We found that genes responsible for the coat colour in the Wuzhishan breed are located on chromosome 8. Ninety-seven genome-wide significant SNPs are related to the animal’s coat colour. Using a haplotype-sharing analysis, we narrowed the potential candidate region to a 10.1 Mb interval encompassing only one gene, *RAPGEF2*, which participates in the regulation of melanogenesis. Two additional candidate genes, *PDGFRA* and *KIT*, are located within 1 Mb of the genome-wide significant SNPs. Gene ontology analysis and literature mining suggest that these candidate genes are associated with the animal’s coat colour. mRNA expression results revealed that *RAPGEF2* and *PDGFRA* had significantly higher expressions in black pigs than in white pigs and higher expressions in black skin than in white skin from the same black-back/white-belly pigs. These results suggest that *RAPGEF2* and *PDGFRA* are potential candidate genes regulating the coat colour in Wuzhishan pigs. Interestingly, mutations of *KIT* (a gene duplication and a G to A substitution at the splicing site in intron 17) were detected in white Wuzhishan pigs but not in black-back/white-belly or black pigs, suggesting a close genetic relationship between white Wuzhishan pigs and Western white pig breeds. In summary, these results indicate that the expression of *RAPGEF2* and *PDGFRA* may cause the coat colour variation by influencing the deposition of melanin, while the mutation of *KIT* causes the white coat colour. Our results may provide a theoretical basis for the breeding of white coat colour Wuzhishan pigs, and shed light on the complex genetic background of coat colour variations in indigenous Chinese pig breeds.

## Simple Summary

The Wuzhishan pig breed exhibits three different coat colour phenotypes (white, black, and black-back/white-belly). The coat colour variation is worth studying. Sixty-one white, 96 black-back/white-belly, and 58 black Wuzhishan pigs were used for genome-wide association analysis. Ninety-seven genome-wide significant SNPs on the animal’s chromosome 8 are related to its coat colour. The *RAPGEF2*, *PDGFRA*, and *KIT* genes were identified as candidate genes for coat colour variation in haplotype-sharing analysis and gene ontology analysis. SNP genotyping and mRNA expression analysis further confirmed *RAPGEF2* and *PDGFRA* as potential candidate genes. *KIT* was confirmed as a potential candidate gene for white coat colour by duplication and splice mutation detection. Our study may also shed light on the complex genetic background of coat colour variation in animals.

## 1. Introduction

One of the most strikingly variable and visible traits that emerged from livestock domestication is the coat colour of livestock. The genetics of coat colour was the focus of pioneering studies in animal genetics at the beginning of the last century [1]. The coat colour is one of the most important characteristics that distinguish pig breeds. The coat colour phenotype of pigs is determined by the relative amounts of eumelanins (black/brown) and pheomelanins (yellow/red) in the pig’s melanocytes. When the synthesis of eumelanin and pheomelanins is blocked, white coat colour phenotypes will be formed. When the synthesis of pheomelanins or eumelanins is blocked, black or other (red, brown, or silver) coat colour phenotypes will be formed. It is now understood that several loci affect this trait in pigs, and numerous studies have shown that genetic factors involved in the coat colour are shared amongst different species [2,3]. Currently, 688 genes are thought to be involved in regulating the coat colour in mice (http://www.espcr.org/micemut (accessed on 3 September 2024)). These coat colour genes influence the development, differentiation, proliferation, and migration of melanocytes, as well as the construction and transport of melanosomes and the synthesis of melanin.

In pigs, variations in several genes affect their pigmentation. Mutations affecting coat colour have been well characterized in the proto-oncogene receptor tyrosine kinase (*KIT*) gene for the dominant white colour, and the melanocortin 1 receptor (*MCIR*) locus has been characterized for coat colour phenotypes such as dominant black, black spots, and red colour [4]. Other genes such as *ASIP*, *TYRP1*, *EDNRB*, *KITLG*, and *OCA2* might also affect coat colour in a few specific breeds or populations, or modify the effects of *KIT* and *MCIR* [4]. Studies of *MC1R* have provided valuable insights into the biology of pigmentation and the evolution of domesticated animals [5,6]. Recent results suggest that the *RAPGEF2* and *PDGFRA* genes are involved in pathways related to *KIT*, and these are well known for their key roles in the underlying mechanisms of coat colour in pigs [7].

Although the major genes that control the coat colour in pigs are now known, they do not fully account for the unusual coat colour characteristics exhibited by Chinese indigenous breeds. First, Chinese pigs show much more diversity in their coat colour than exotic pigs. Long-term natural selection and artificial breeding in different regions have resulted in phenotypes such as solid black, white [8], two-end black [9], brown [10], belted and spotted, and several other different phenotypes. Second, even when their phenotypes are identical, Chinese and exotic pigs’ underlying genetics can differ. For example, the white colour in introduced pigs, including Yorkshire and Landrace pigs, is controlled by mutations in *KIT* [11]. However, these mutations are not found in Chinese Rongchang pigs, which are also white [8]. Finally, although most pig breeds exhibit a single characteristic coat colour, some Chinese breeds vary in colour within their breed due to relaxed selection and wide geographic distribution. For example, Tibetan pigs in the Qinghai-Tibetan Plateau have black, reddish, and silver-brown coat colours. Kele and Dahe pigs in the Yunnan-Guizhou plateau have coats that range from light to dark brown [10]. The classic Wuzhishan pig is characterized by a black-back/white-belly phenotype, with additional black and white variants, thus providing an ideal model for investigating the genetic variation in coat colour in pigs.

Although within-breed colour variations are potentially complex, they facilitate using a genome-wide association study (GWAS) to locate loci which control that breed’s coat colour. We used a GWAS to identify candidate genes for genetic variations that could be associated with the three different coat colours in Wuzhishan pigs. The results provide clues to understanding the genetic factors that control the coat colour in Chinese indigenous pigs.

## 2. Materials and Methods

### 2.1. Animal Sampling and Phenotyping

**Figure 1:**
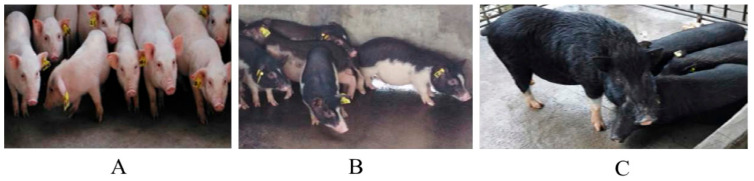
Three coat colour phenotypes in Wuzhishan pigs. (**A**) white, (**B**) black-back/white-belly, (**C**) black.

Samples were obtained from 215 Wuzhishan pigs at the Institute of Animal and Veterinary Sciences at the Hainan Academy of Agricultural Sciences (Haikou, China). Individual phenotypes were determined by direct visual inspection or from photographs taken outdoors on a sunny day, and recorded for use in the genome-wide association (GWA) analysis. Animals were classified into one of three categories based on coat colour: white (*n* = 61), black-back/white-belly (*n* = 96), and black (*n* = 58) (Figure 1). For ear tissue collection, the sampling area was first cleaned with 75% alcohol, and approximately 10 g of tissue was removed using clean scissors and stored at −20 °C until use. Twelve skin samples were obtained from 3 white pigs, 3 black pigs, and 3 black-back/white-belly pigs (both belly and back skin was sampled) and stored at −80 °C until use. The wound was treated with iodine tincture.

### 2.2. Genotyping and Quality Control

Genomic DNA was extracted from ear tissues using the phenol-chloroform method [12]. To ensure successful genotyping, the quality and quantity of extracted DNA was checked with NanoDrop™ 2000 (Thermo Fisher Scientific Inc., Waltham, MA, USA). DNA samples were retained only if concentration exceeded 50 ng/μL, total volume exceeded 50 μL, and light absorption ratio (A260/280) was between 1.8 and 2.0. Genotyping was conducted by Neogen (Shanghai, China) using the GeneSeek-Neogen PorcineSNP80 BeadChip (GeneSeek, Lincoln, NE, USA). Genotypes were assigned using GenomeStudio (https://www.illumina.com/techniques/microarrays/array-data-analysis-experimental-design/genomestudio.html (accessed on 5 June 2024)).

To reduce false-positive associations, we required a genotyping call rate threshold ≥ 90%, a Hardy–Weinberg equilibrium *p*-value ≥ 10^−6^, and a minor allele frequency ≥ 3% (parameters set in PLINK). All SNPs for which the chromosomal location could not be determined were removed from the analysis. The resulting data set contained 40,345 markers.

### 2.3. Genome-Wide Association Study

A single-marker GWAS was conducted using the general linear model Y = Xβ + e [13]; specifically, Y = μ + SNPi + b1 PC1 + b2 PC2 + b3 PC3 + e. Y represents the phenotype (white = 0, black-back/white-belly = 1, black = 2), μ represents the phenotypic mean, and SNPi represents the genotype of the ith SNP (AA = 0, Aa = 1, aa = 2). PC1, 2, and 3 represent the three principal components calculated by the EIGENSTRAT program and are used to correct for population stratification of the phenotypic data. e represents residual error, and b represents covariance regression coefficient. No fixed effects were included in the model because all pigs were from the same farm and because coat colour does not change with age nor is it affected by gender. Bonferroni multiple testing was used to correct for genome-wide significance (*p*-value/N), where N is the number of SNPs used for analyses. Because there were 40,345 SNPs in the analysis, we set the *p*-value threshold as 10^−6^. The quantile–quantile (Q–Q) plot, a commonly used tool for visualizing population stratification in GWASs, was utilized in our analysis to test the effect of correcting the population stratification with the principal components and to identify significant SNPs. Reconstruction of haplotypes in the mapped interval was performed with Haploview 4.2 (https://www.broadinstitute.org/haploview/haploview (accessed on 3 August 2024)) to identify genes related to coat colour. Fixation index (FST) estimates between population pairs were calculated for all SNPs based on Weir and Cockerham statistics using VCFtools 4.2 [14]. Average FST was then calculated for each 500 kb window with 200 kb overlapping adjacent windows. FST plots were generated with these FST values for each population pair.

### 2.4. Gene Ontology Analysis

To identify coat colour-related candidate genes, genes within 1 Mb regions surrounding significant SNPs were identified using the pig genome sequence (Sscrofa 11.1 genome version) in Ensembl (https://useast.ensembl.org/Sus_scrofa/Info/Index (accessed on 2 December 2024)). To infer function for unannotated genes, we used their IDs to query Ensembl BioMart (http://useast.ensembl.org/biomart/martview/eaf4bd4a354980cb5dcdc7a0516c50be (accessed on 2 December 2024)) to obtain homologous human genes. Gene ontology analysis was carried out using kobas2.0 (http://bioinfo.org/kobas/annotate/ (accessed on 2 December 2024)).

### 2.5. Detection of Duplications and Splice Mutations in the KIT Gene

Marklund et al. have reported that duplication or a G to A substitution at the first nucleotide in intron 17 of the *KIT* gene causes a dominant white phenotype in exotic pigs [11]. These mutations, however, are absent from the white-coated Rongchang pig breed [8]. To determine whether these mutations exist in the Wuzhishan pig population, DNA from all the Wuzhishan pig samples, along with DNA from Yorkshire, Landrance, (positive controls), and Rongchang pigs (negative control) was tested as described previously [15] for the duplication/substitution in the *KIT* gene. Primers (Table 1) were designed so that a 152 bp fragment spanning the duplication breakpoint could only be amplified by a template of the duplication. The thermal cycle conditions were 95 °C for 5 min, followed by 35 cycles at 95 °C for 30 s, 55 °C for 30 s, 72 °C for 30 s, and, finally, 72 °C for 10 min.

To detect the presence of the G to A substitution, primers (Table 1) were designed to amplify a 175 bp fragment spanning the boundary between exon 17 and intron 17. The cycling conditions were: 95 °C for 5 min, followed by 35 cycles at 95 °C for 30 s, 60 °C for 30 s, 72 °C for 30 s, and, finally, 72 °C for 10 min. The PCR products from pigs of the same coat colour were pooled and then sequenced by the Beijing Genomics Institute (Beijing, China). To further verify this unique mutation in white pigs, three individuals were randomly selected from each of the three colour groups, and the same 175 bp PCR products for each were digested overnight at 37 °C with restriction endonuclease NlaIII (New England BioLabs, Ipswich, MA, USA), which cuts once in the wild type sequence but twice in the mutant sequences. In these experiments, DNA from Yorkshire and Rongchang pigs was used as positive and negative controls, respectively.

### 2.6. Real-Time Quantitative PCR (RT-qPCR) Analysis

Twelve skin samples from 3 white pigs, 3 black pigs, and 3 black-back/white-belly pigs (both belly and back skin were sampled) were collected for RNA isolation. From each pig, a skin punch biopsy that had a diameter of 1 cm and a thickness of 4 cm was collected from the abdomen or the back. All procedures were done under local anaesthesia at the Institute of Animal and Veterinary Sciences at the Hainan Academy of Agricultural Sciences (Haikou, China). Total RNA was isolated with TRIzol reagent (Invitrogen, Carlsbad, CA, USA) following the manufacturer’s protocol, and 1 ug of RNA was used for cDNA synthesis using the FastQuant RT kit (Tiangen, Beijing, China) and following the manufacturer’s protocol.

RT-qPCR analysis was performed to assess the expression of *RAPGEF2* and *PDGFRA* (GenBank accession numbers: XM_021101305 and NM_001315756, respectively) mRNA in white and black skin (primers in Table 1). Amplification was conducted as previously described [16], and the 2^−ΔΔCt^ method was used to analyse gene expression levels [17]. *GAPDH* (GenBank Accession number: NM_001206359) was the internal control (primers in Table 1).

### 2.7. Statistical Analysis

The statistical analysis results are shown as the means ± standard error (SE). The significant differences among the groups were identified through one-way analysis of variance (ANOVA). The different groups were compared using Duncan’s new multiple range test in the R package of Agricolae (version 1.2-8), R version 3.3.3. The differences were considered statistically significant at *p*-values < 0.05.

## 3. Results

### 3.1. Descriptive Statistics for SNPs

Out of 80,000 SNPs in the initial data set, 68,516 SNPs remained after the quality control criteria were applied. An additional 25,304 SNPs were removed by PLINK, leaving 43,212. A total of 2867 SNPs could not be assigned to a chromosomal location and were also removed, leaving 40,345 for the GWA analysis. The average physical distance between two neighbouring SNPs on the same chromosome was approximately 60.7 Kb, and ranged from 38.4 Kb on SSC12 to 77.4 Kb on SSCX. Based on the length of each chromosome in the USDA-MARC v2 (A) linkage map (http://www.thearkdb.org/), the genetic distances between adjacent SNPs on the SNP chip ranged from 0.0819 cM (SSC17) to 0.0339 cM (SSC1), with an average of 0.0575 cM (Table S1).

### 3.2. GWAS

**Figure 2:**
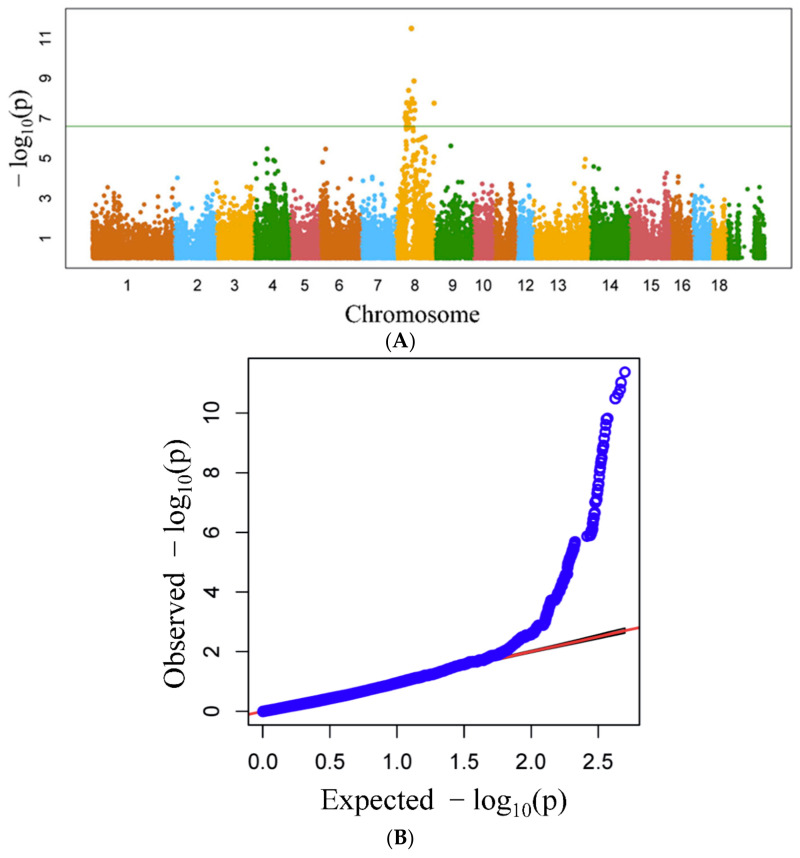
(**A**) Manhattan plot showing results from the genome-wide association study for coat colour traits in Wuzhishan pigs. (**B**) Quantile–quantile plot showing observed (black line) versus expected (blue points) log10 (*p*-values). The null hypothesis (no association between SNPs and coat colour) is represented by the red line.

**Figure 3:**
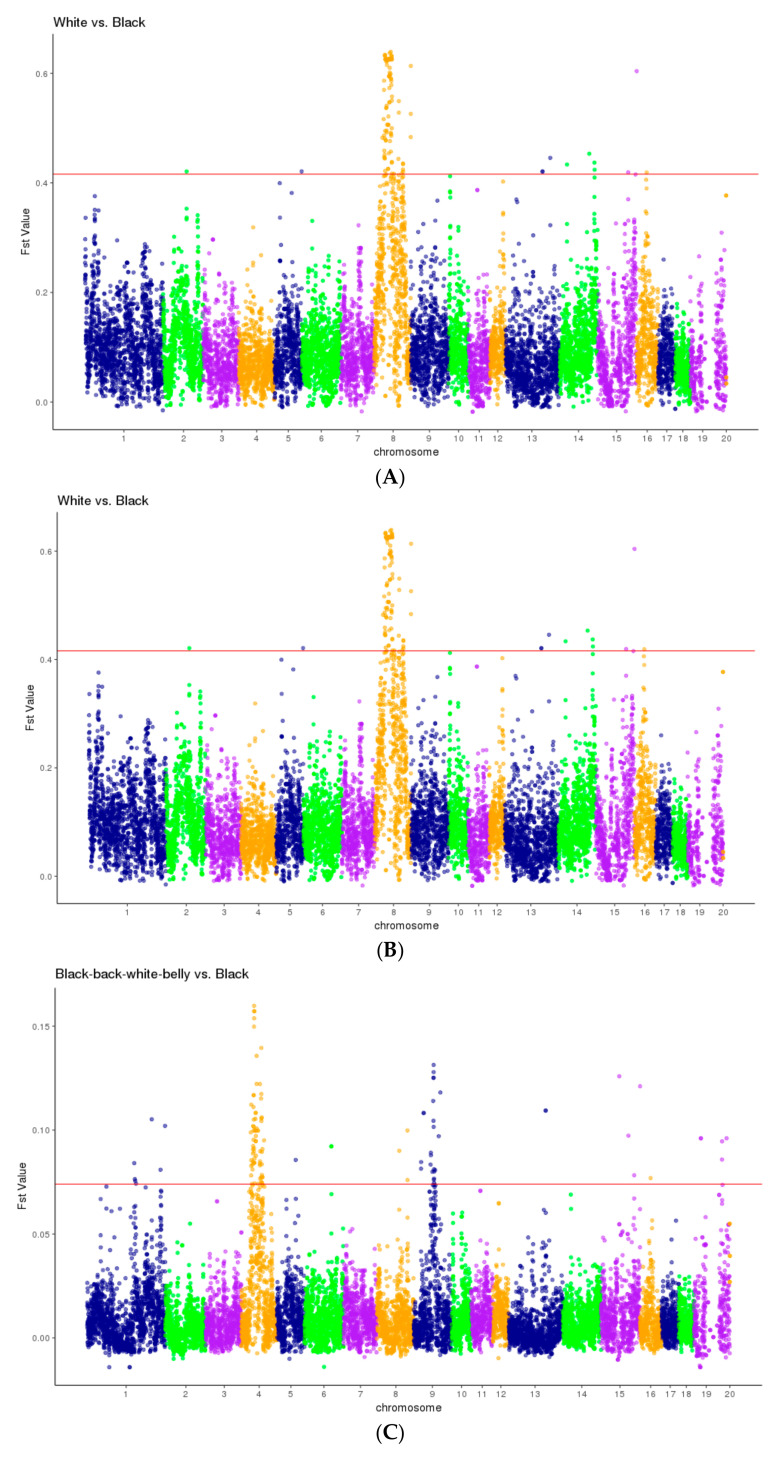
Fixation index (FST) plots generated using average FST values, with 500 Kb windows and 200 Kb overlap between adjacent windows. (**A**) FST plot for white vs. black. (**B**) FST plot for white vs. black-back/white-belly. (**C**) FST plot for black vs. black-back/white-belly. The horizontal red line indicates the top 1% FST values. SNPs with values above this threshold were considered to be selective sweep loci.

The significances (–log10[*p*-value]) of associations between the SNPs and coat colour traits were calculated and the results are shown in Figure 2. The Manhattan plot (Figure 2A) shows statistically significant (defined as –log10[*p*-value] ≥ 6) associations between coat colour and 97 SNPs spanning a 43 Mb region (Table S2) on pig chromosome 8 (SSC8). The Q–Q plot in Figure 2B shows the deviation between the observed and expected *p*-values, based on the null hypothesis of no true association between SNPs and coat colour. The analysis indicates that a strong association exists between some SNPs and coat colour. Genome-wide FST plots revealed a significantly high genetic differentiation (FST values above the 1% threshold, i.e., >0.4) between the white and black, and white and black-back/white-belly populations (Figure 3A and 3B, respectively) at the 43 Mb region, which lies within the 62 Mb positive selective sweep region (ssc8: 31982533-93688528). However, the FST between the black and black-back/white-belly populations is mostly much lower, with a 1% threshold of around 0.07 and a maximum of around only 0.15 (Figure 3C), indicating a small genetic distance between these two populations.

**Figure 4:**
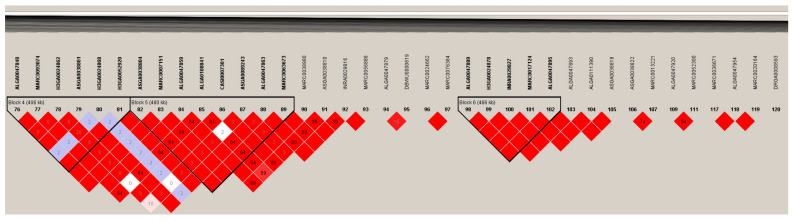
Linkage disequilibrium (LD) plot generated by Haploview4.2 for SNPs in the 10.1 Mb region between the two most significant SNPs (DRGA0008593 and ALGA0047848) in SSC8. SNP IDs are indicated horizontally across the top. The black lines indicate the identified haplotype blocks containing significant SNPs with complete linkage disequilibrium. Red diamonds represent LD between two SNPs with r^2^ values lower than 100% when labelled but that are equal to 100% when unlabelled.

Among the identified significant SNPs on SSC8, the one with the strongest association with coat colour is DRGA0008593 at 57.77 Mb (*p*-value = 3.23 × 10^−12^), followed by ALGA0111438 (*p*-value = 1.37 × 10^−9^) at 66.23 Mb and ALGA0047848 (*p*-value = 4.01 × 10^−9^) at 47.69 Mb. Linkage disequilibrium analysis of the 97 genome-wide significant SNPs showed nine haplotype blocks with complete linkage disequilibrium. Among these, blocks 4, 5, and 6 are located in the 10.1 Mb region between DRGA0008593 and ALGA0047848 (Figure 4). Interestingly, block 4 (406 Kb) and block 5 (408 Kb) are close to each other, and block 4 covers the most significant SNP, ALGA0047848 (Figure 4). Nine additional SNPs are found in these two blocks (MARC0093074 at 47.87 Mb, ASGA0038801 at 48.00 Mb, MARC0007151 at 48.16 Mb, H3GA0052920 at 48.10 Mb, ASGA0038804 at 48.13 Mb, ALGA0047859 at 48.21 Mb, CASI0007301 at 48.32 Mb, ALGA0047863 at 48.50 Mb, and MARC0063673 at 48.70 Mb). Together, these observations strongly suggest that one or more genes related to coat colour are within these blocks

The SNPs in blocks 4 and 5 implicate the only two genes that are located in these blocks: *C8H4orf45*, an unannotated gene, and *RAPGEF2* (rap guanine nucleotide exchange factor 2) (Table S2). Using the pig reference genome assembly, we searched for annotated genes that were ±1 Mb from each significant SNP (Table S2), and found that most were not reported to be associated with coat colour. Two additional genes that are potentially associated with coat colour were identified by gene ontology analysis (using kobas2.0) and literature mining: *PDGFRA* (platelet-derived growth factor receptor alpha) is associated with melanocyte defects, and *KIT* (mast/stem cell growth factor receptor) has already been identified as the dominant white locus. Therefore, we selected *RAPGEF2* and *PDGFRA* as potential candidate genes and investigated whether their SNPs or expression differ between Wuzhishan pigs with different coat colour phenotypes. We also investigated the effect of *KIT* in our experimental population.

### 3.3. SNPs Genotyping Adjacent to RAPGEF2 and PDGFRA

Five SNPs adjacent to *RAPGEF2* (ASGA0038801, H3GA0052920, ASGA0038804, MARC0007151, and ALGA0047859) and one adjacent to *PDGFRA* (WU_10.2_8_43364095) became a focus of this study. After genotyping all the pigs at these SNPs (Table 2), we found that they are tightly linked into two haplotypes. Despite the genotyping failure of one sample at MARC0007151 and ALGA0047859, it is apparent that white pigs are all heterozygotes or homozygotes of one haplotype and that black and black-back/white-belly pigs are all homozygotes of the other haplotype.

### 3.4. Expression of RAPGEF2 and PDGFRA in Different Skins and Pigs

**Figure 5:**
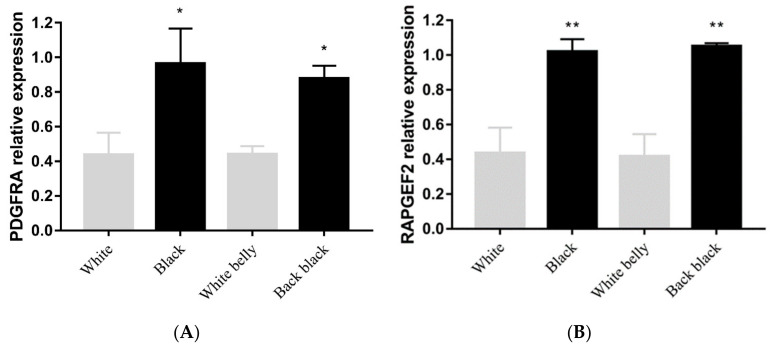
Expression of (**A**) *RAPGEF2* and (**B**) *PDGFRA* in skin tissues from Wuzhishan pigs exhibiting one of the three possible coat colours. Results are shown as means ± SD of triplicate measurements. * indicates *p* < 0.05, ** indicates *p* < 0.01. White belly and black back are both samples from black-back/white-belly pigs.

Because both black-back/white-belly and black Wuzhishan pigs have the same genotype at the six SNPs near *RAPGEF2* and *PDGFRA*, we investigated the expression of mRNA from these genes in Wuzhishan pigs of all three possible coat colours. RT-qPCR showed that *RAPGEF2* and *PDGFRA* not only had significantly higher expressions (*p* < 0.01) in black pigs than in white pigs, but that they also had higher expressions (*p* < 0.05) in black skin than in white skin from the same black-back/white-belly pigs. However, there was no significant difference in the expression of these genes between samples of the same colour of skin taken from white pigs or black pigs and black-back/white-belly pigs (Figure 5A,B).

### 3.5. Detection of Duplication and Splice Mutation in KIT Gene

Compared to *RAPGEF2* and *PDGFRA*, another gene of interest—the *KIT* gene—is very well-known for its duplication and splice mutation leading to the white coat phenotype in Western pigs [18]. However, such a mutation is absent in a Chinese white pig breed—Rongchang pigs [8]. Therefore, we did PCR to examine whether the white coat colour in Wuzhishan pigs is associated with *KIT* gene duplication. The PCR product of the 152 bp fragment spanning the duplication breakpoint in the *KIT* gene was only observed in the 30 white Wuzhishan pigs and the positive controls—Yorkshire and Landrace—but not in the black–back-white-belly or black Wuzhishan pigs or the negative control—Rongchang pig (Figure S1), indicating that, unlike Rongchang pigs, *KIT* gene duplication is associated with white coat colour in Wuzhishan pigs.

**Figure 6:**
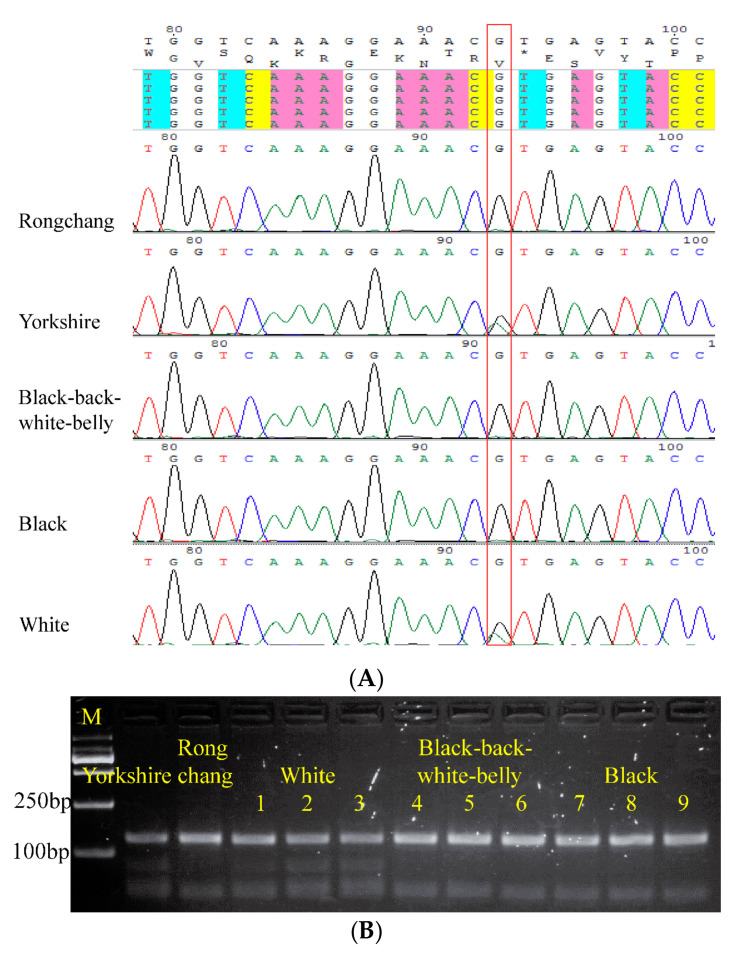
The G to A mutation located at the first nucleotide in intron 17 of *KIT*, which causes alternative splicing, was detected through sequencing and restriction digestion. (**A**) Sequence alignment of data obtained from pooled DNA samples (from 30 individuals) shows the mutation in *KIT* intron 17. The red column highlights the mutation site (93G > A); Yorkshire and white Wuzhishan pigs exhibit double peaks, indicating nucleotides G and A at that location, while all the other pigs showed single peaks at this location, indicating the presence of only G nucleotide. (**B**) Agarose gel displaying the PCR product from *KIT* intron 17 of Wuzhishan pigs after digestion with NlaIII. Lanes 1–3 contain DNA from white pigs, 4–6 from black-back/white-belly pigs, and 7–9 from black pigs. The three bands in the Yorkshire and white pig lanes are diagnostic of the splice mutation, while the double bands are wild type. The Yorkshire and Rongchang pigs served as positive and negative controls, respectively.

In addition to its duplication, the *KIT* gene has another G to A mutation at intron 17, which leads to the loss of exon 17 in transcripts and thus a white coat colour. Direct sequencing of the 175 bp fragments spanning this SNP showed the presence of a nucleotide only in the white pigs among the three coat colour groups of Wuzhishan pigs, which is also true for Yorkshire pigs (Figure 6A). But all the other pigs showed only the presence of G nucleotide, in which is also true for Rongchang pigs. Digestion of the 175 bp fragments with restriction endonuclease NlaIII confirmed this result, with white Wuzhishan pigs and the Yorkshire pig showing three bands in the gel electrophoresis after digestion, while the black and black-back/white-belly pigs and the Rongchang pig showed two bands (Figure 6B). Therefore, our results indicate that, compared to Rongchang pigs, Wuzhishan pigs may have a closer genetic relationship with exotic pigs, with a white coat colour being strongly related to the two mutations in the *KIT* gene [11].

## 4. Discussion

In domestic animals, GWA analyses based on large-scale SNP scans provide a powerful tool for identifying genes or mutations that underlie phenotypic traits [19]. In this study, we performed a GWA analysis to scan loci associated with the coat colour in Wuzhishan pigs. All of the 97 significant SNPs identified by the GWAS were located in a 43 Mb region on chromosome 8, which is covered by a positive selective sweep region that differentiates between white, black, and black-back/white-belly pigs, as revealed by FST analysis. Using the three most significant SNPs and linkage disequilibrium analysis, we narrowed our focus to a 10.1 Mb region that contained *RAPGEF2*. In addition, *KIT* and *PDGFRA* were also found in the 1 Mb regions flanking some SNPs, but only *KIT* is known to be a regulator for a white coat colour in pigs. Because *RAPGEF2* and *PDGFRA* were related to the coat colour in pigs [7,20] and mice [21,22], they are potential candidates for regulating coat colour variation in Wuzhishan pigs.

*RAPGEF2*, a member of the Ras subfamily of GTPases, augments cAMP-mediated Ras/ERK activation, thereby enhancing the movement of melanosomes to newly formed dendrites, melanin production, and secretion. The high expression of *RAPGEF2* also regulates melanocyte survival [21,23,24]. Amsen et al. observed a high expression of *RAPGEF2* in B16 melanoma cells [25], which is consistent with the higher expression of *RAPGEF2* in the black skin from Wuzhishan pigs than in the white skin (Figure 5A). In addition, *RAPGEF2* genes were also involved in the pathways related to *KIT*, *PDGFRA*, or *KDR* genes, which comprise a classical cluster of tyrosine kinase receptors in cattle’s coat colour [7,26]. Therefore, *RAPGEF2* has reason to be considered as a putative candidate gene for influencing the coat colour in Wuzhishan pigs.

*PDGFRA* encodes a tyrosine kinase receptor, and its activation is important for deriving melanocytes from the neural crest [27]. A deletion encompassing *PDGFRA* is found in Patch (Ph) mice, which exhibit defects of neural crest origin that affect melanocyte migration, even in heterozygotes, resulting in a white patch on the mouse’s trunk [22]. *PDGFRA* mutation also results in melanocyte deficiencies, suggesting that *PDGFRA* may function during early pig development by regulating melanocyte differentiation and proliferation [28,29,30,31]. The potential involvement of *PDGFRA* in melanocyte development in pigs is consistent with the significantly higher expression of *PDGFRA* observed in black skin vs. white skin from Wuzhishan pigs (Figure 5B). The close proximity of *PDGFRA* to *KIT* on the short arm of Wuzhishan pig chromosome 8 also suggests that *PDGFRA* indirectly regulates the coat colour through interaction with *KIT* [3,7]. In recent years, *PDGFRA* has been reported as a putative candidate gene for the coat colour in pigs [7], cattle [32,33], and goats [34,35]. The data, therefore, support *PDGFRA* as a potential candidate gene regulating the coat colour phenotype in Wuzhishan pigs.

The *KIT* gene plays a key role in different coat colour patterns in pigs [7,36], cats [37,38], cattle [32,39], mice [40,41], and goats [35,42]. Porcine *KIT* is known to be a dominant white locus, and mutations in *KIT* affect the coat colour and colour distribution in exotic pigs. However, mutations in this gene have not been found in the white-coated Rongchang pig, a Chinese indigenous breed, suggesting an alternative genetic cause for the white coat colour in these pigs. This difference may also reflect independent evolution or breeding of the Rongchang pig from exotic breeds. This study found both the duplication and the G to A substitution at the splicing site in intron 17 of *KIT* in white Wuzhishan pigs, but not in black-back/white-belly or black pigs (Figure S1 and Figure 6). Therefore, it is likely that the white coat colour in the Wuzhishan pig population is highly associated with mutations of *KIT*, suggesting a close genetic relationship between the white Wuzhishan pig and white exotic breeds such as Yorkshire and Landrace.

The development of hair follicles and melanocytes is a complex event involving numerous interacting genes and pathways. Our GWAS and validation results showed that *KIT* mutations are highly associated with white coat phenotypes in Wuzhishan pigs. *RAPGEF2* and *PDGFRA* are also candidate genes for white coat colour loci in indigenous Chinese pig breeds, such as the Wuzhishan pig, but their presumed functions are based largely on data obtained from mice. Further study, such as gene editing studies, will be needed to clarify the role of these genes in the coat colour phenotypes of pigs, [43,44].

## 5. Conclusions

Our genome-wide association study and expression analysis showed that *KIT* is an important gene that is associated with the white coat colour variance in the Wuzhishan pig population, and that *RAPGEF2* and *PDGFRA* may also be potential candidate genes that play an important role in the coat colour variation in Wuzhishan pigs. In summary, our results suggest that the mutation of *KIT* causes the Wuzhishan pig’s white coat colour, while the expression of *RAPGEF2* and *PDGFRA* may cause the Wuzhishan pig’s coat colour variation by influencing the deposition of melanin. Our study may provide a theoretical basis for the breeding of white coat colour Wuzhishan pigs, and also shed light on the complex genetic background of coat colour variation in animals.

## Figures and Tables

**Table 1:** The primers for PCR and RT-qPCR.

Gene	Length	Primers (5′-3′)	Annealing Temperature
***KIT*** (PCR)	152 bp	F: TAAGTGAAAGAAGTCAATCTGAG R: GGCAGTCATGTAACTATCACC	55 °C
***KIT*** (PCR)	175 bp	F: GTATTCACAGAGACTTGGCGGC R: AAACCTGCAAGGAAAATCCTTCACGG	60 °C
***RAPGEF2*** (RT-qPCR)	223 bp	F: GCAGTCCCACCATCGCAT R: AGTCACAGCAAACTCCCG	60 °C
***PDGFRA*** (RT-qPCR)	151 bp	F: CTTGGGGTTGAGAGCCGA R: TTTCATACCTGGGTTTCTGTTTC	60 °C
***GAPDH*** (RT-qPCR)	170 bp	F: GGTCGGAGTGAACGGATTTG R: CCTTGACTGTGCCGTGGAAC	60 °C

**Table 2:** Genotype of SNPs adjacent to *RAPGEF2* and *PDGFRA*.

SNP ID	SSC ^1^ & Position ^2^	Closest Adjacent Gene	Genotype	White Number	BBWB ^3^ Number	Black Number
ASGA0038801 (G/A)	SSC8:48005002	*RAPGEF2*	AA	20	0	0
			GA	41	0	0
			GG	0	96	58
H3GA0052920 (A/G)	SSC8:48096551	*RAPGEF2*	GG	20	0	0
			GA	41	0	0
			AA	0	96	58
ASGA0038804 (C/T)	SSC8:48130141	*RAPGEF2*	TT	20	0	0
			TC	41	0	0
			CC	0	96	58
MARC0007151 (T/C)	SSC8:48155658	*RAPGEF2*	CC	20	0	0
			CT	40	0	0
			TT	0	96	58
ALGA0047859 (A/G)	SSC8:48209595	*RAPGEF2*	GG	20	0	0
			GA	40	0	0
			AA	0	96	58
WU_10.2_8_43364095 (T/C)	SSC8:41273714	*PDGFRA*	CC	20	0	0
			CT	41	0	0
			TT	0	96	58

^1^ Su^1^s scrofa chromosome; ^2^ Derived from the porcine genome sequence assembly Sscrofa11.1; ^3^ black-back/white-belly Wuzhishan pigs.

## Data Availability

The datasets presented in this study can be found in online repositories. The names of the repository/repositories and accession numbers can be found at https://doi.org/10.6084/m9.figshare.27674208.v1 (accessed on 17 November 2024).

## Supplementary Materials

The following supporting information can be downloaded at: https://www.mdpi.com/article/10.3390/ani14233493/s1, Table S1. Number and size of SNP loci after quality control, and average distance between adjacent SNPs on each chromosome, Table S2. Genome-wide survey of significant SNPs associated with coat colour and adjacent genes, Figure S1. Agarose gel displaying the PCR product amplified from *KIT*.
