# Supplementary material for: Discovery of Potential Candidate Genes for Coat Colour in Wuzhishan Pigs by Integrating SNPs and mRNA Expression Analysis
Source: Animals (Basel). 2024 Dec 3;14(23):3493. doi: 10.3390/ani14233493 (PMC11640690; doi:10.3390/ani14233493)
Supplement: Supplementary file 1 [file animals-14-03493-s001.zip › animals-3293117-supplementary.pdf]

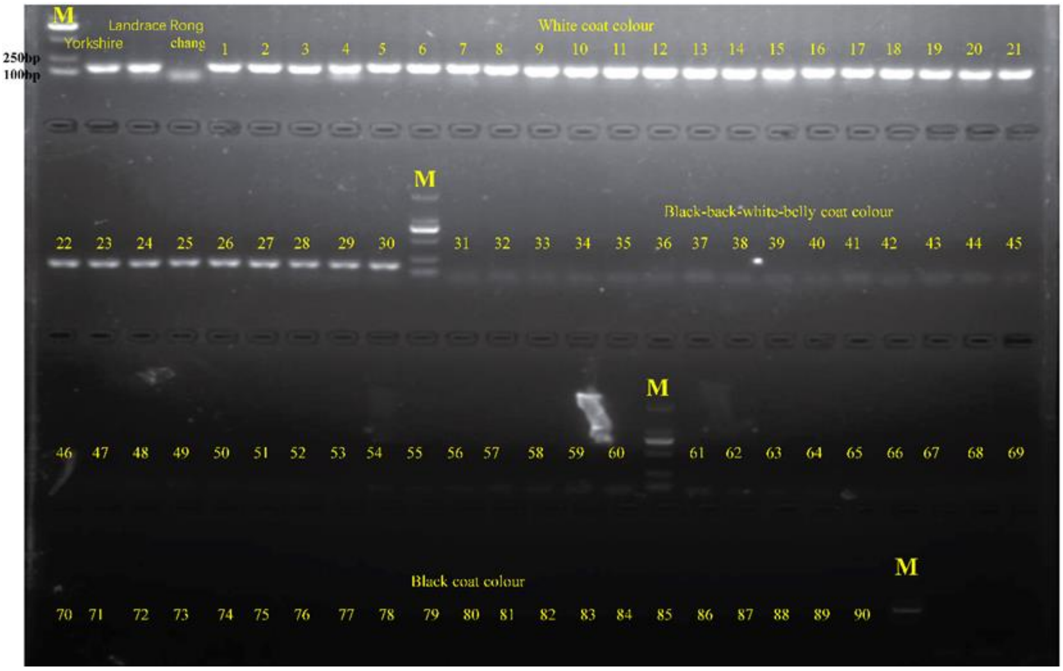

Figure S1. Agarose gel displaying the PCR product amplified from *KIT* in white (1-30), black-back/white-belly (31-60), and black (61-90) Wuzhishan pigs. M indicates lanes containing size markers. Samples with two copies of *KIT* show a bright band at 152 bp. Yorkshire and Landrace are positive controls and Rongchang is a negative control.

Table S1. Number and size of SNP loci after quality control, and average distance between adjacent SNPs on each chromosome.

SSC	SNP no.	Size (Mb)[1]	Mb/SNP	Linkage distance (cM)[2]	cM/SNP
1	4247	315.32	0.0742	144.0	0.0339
2	2764	162.34	0.0587	132.1	0.0478
3	2231	144.65	0.0648	129.3	0.0580
4	2431	143.47	0.0590	130.1	0.0535
5	1716	111.51	0.0650	114.4	0.0667
6	2585	157.76	0.0610	165.7	0.0641
7	2587	134.76	0.0521	156.6	0.0605
8	2484	148.49	0.0598	127.7	0.0514
9	2013	153.66	0.0763	138.5	0.0688
10	1782	78.83	0.0442	124.1	0.0696
11	1596	87.67	0.0549	84.9	0.0532
12	1656	63.58	0.0384	113.1	0.0683

13	2863	218.61	0.0764	126.2	0.0441
14	2828	153.84	0.0544	111.5	0.0394
15	2037	157.62	0.0774	111.8	0.0549
16	1424	86.89	0.0610	93.2	0.0654
17	1184	69.36	0.0586	97.0	0.0819
18	1072	61.20	0.0571	57.6	0.0537

SNP, single nucleotide polymorphisms; SSC, *Sus scrofa* chromosome; [1]The physical size is based on *Sus scrofa* Build 10.2 (http://www.ensembl.org/Sus_scrofa/Info/Index); [2]The linkage distance is based on USDA-MARC v2 (A) (http://www.thearkdb.org/); There are 40435 total SNPs, of which 845 are unmapped.

Table S2. Genome-wide survey of significant SNPs associated with coat color and adjacent genes.

SNP ID	SSC[1]	Position[2]	p-value	Closet adjacent gene	Gene ID	Distance	Gene location
DRGA0008593	8	57766670	3.23E-12	NA			
ALGA0111438	8	66234781	1.37E-09	*UGT2B31*	ENSSSCG00000039276	75916	ssc8: 66310697-66323755
ALGA0047848	8	47692165	4.01E-09	*PPID*	ENSSSCG00000008878	within	ssc8: 47656269-47702805
ALGA0047974	8	59859539	1.05E-08	*ADGRL3*	ENSSSCG00000026129	128741	ssc8: 59988280-60800177
ALGA0047809	8	41806441	1.64E-08	*KDR*	ENSSSCG00000008844	2675	ssc8: 41809116-41856379
WU_10.2_8_42826282	8	40783941	1.64E-08	*CHIC2*	ENSSSCG00000032645	within	ssc8: 40781365-40846715
ALGA0112291	8	40498539	1.71E-08	*LNX1*	ENSSSCG00000008838	25438	ssc8: 40232837-40473101
MARC0039159	8		1.76E-08				
MARC0075425	8	44711400	1.76E-08	*TDO2*	ENSSSCG00000008868	within	ssc8: 44708722-44725954
WU_10.2_8_46863216	8	44922716	1.76E-08	*CTSO*	ENSSSCG00000008867	157353	ssc8: 44727626-44765363
MARC0029724	8	44999262	1.76E-08	*CTSO*	ENSSSCG00000008867	233899	ssc8: 44727626-44765363
ASGA0038801	8	48005002	1.76E-08	*RAPGEF2*	ENSSSCG00000008881	178516	ssc8: 48183518-48277476
MARC0007151	8	48155658	1.76E-08	*RAPGEF2*	ENSSSCG00000008881	27860	ssc8: 48183518-48277476
ALGA0047863	8	48506057	1.76E-08	*RAPGEF2*	ENSSSCG00000008881	228581	ssc8: 48183518-48277476
INRA0029816	8	48938527	1.76E-08	*RAPGEF2*	ENSSSCG00000008881	661051	ssc8: 48183518-48277476
ALGA0047889	8	52045174	1.76E-08	*NAF1*	ENSSSCG00000008887	32356	ssc8: 51972288-52012818
INRA0029827	8	52205338	1.76E-08	*NPY5R*	ENSSSCG00000008889	22002	ssc8: 52173906-52183336
ALGA0047895	8	52458798	1.76E-08	*MARCH1*	ENSSSCG00000039175	within	ssc8: 52375510-52951031

ALGA0111390	8	52733998	1.76E-08	*MARCH1*	ENSSSCG00000039175	within	ssc8: 52375510-52951031
MARC0052300	8	55450942	1.76E-08	*KIAA1211*	ENSSSCG00000008903	within	ssc8: 55336363-55600931
ALGA0047954	8	56983392	1.76E-08	*IGFBP7*	ENSSSCG00000008913	625570	ssc8: 56283089-56357822
MARC0020164	8	57077360	1.76E-08	*IGFBP7*	ENSSSCG00000008913	719538	ssc8: 56283089-56357822
ALGA0047965	8	59670278	1.76E-08	*ADGRL3*	ENSSSCG00000026129	318002	ssc8: 59988280-60800177
H3GA0024924	8	63610818	1.76E-08	*EPHA5*	ENSSSCG00000008919	6084	ssc8: 63285006-63604734
ASGA0085363	8	63883203	1.76E-08	*5S_rRNA*	ENSSSCG00000021957	274111	ssc8: 64157314-64157411
ASGA0038876	8	63983448	1.76E-08	*5S_rRNA*	ENSSSCG00000021957	173866	ssc8: 64157314-64157411
DRGA0008614	8	64237553	1.76E-08	*5S_rRNA*	ENSSSCG00000021957	80142	ssc8: 64157314-64157411
ALGA0048042	8	64283952	1.76E-08	*5S_rRNA*	ENSSSCG00000021957	126541	ssc8: 64157314-64157411
ISU10000067	8	65489709	1.76E-08	*UBA6*	ENSSSCG00000008924	18880	ssc8: 65381058-65470829
DRGA0008622	8	65550880	1.76E-08	*TMPRSS11D*	ENSSSCG00000008926	19553	ssc8: 65570433-65623785
MARC0030810	8	67497387	1.76E-08	*RUFY3*	ENSSSCG00000025514	within	ssc8: 67494552-67582144
WU_10.2_8_71775345	8	67906481	1.76E-08	*SLC4A4*	ENSSSCG00000008943	within	ssc8: 67768955-68177213
ALGA0109302	8	67975238	1.76E-08	*SLC4A4*	ENSSSCG00000008943	within	ssc8: 67768955-68177213
WU_10.2_8_148406495	8	66971500	1.76E-08	*CSN1S2*	ENSSSCG00000009263	within	ssc8: 66971081-67012343
WU_10.2_8_148483193	8	67094339	1.76E-08	*ODAM*	ENSSSCG00000009266	26222	ssc8: 67059284-67068117
M1GA0011935	8	45378964	1.76E-08	*PDGFC*	ENSSSCG00000024960	200101	ssc8: 45579065-45808789
INRA0029810	8	46773019	1.76E-08	*FAM198B*	ENSSSCG00000008873	263886	ssc8: 47036905-47133914
MARC0085941	8		1.76E-08				
MARC0093074	8	47868597	1.76E-08	*C4orf45*	ENSSSCG00000037757	within	ssc8: 47845331-47944239
H3GA0052920	8	48096551	1.76E-08	*RAPGEF2*	ENSSSCG00000008881	86967	ssc8: 48183518-48277476
ASGA0038804	8	48130141	1.76E-08	*RAPGEF2*	ENSSSCG00000008881	53377	ssc8: 48183518-48277476
ALGA0047859	8	48209595	1.76E-08	*RAPGEF2*	ENSSSCG00000008881	within	ssc8: 48183518-48277476
CASI0007301	8	48319713	1.76E-08	*RAPGEF2*	ENSSSCG00000008881	42237	ssc8: 48183518-48277476
MARC0063673	8	48696402	1.76E-08	*RAPGEF2*	ENSSSCG00000008881	418926	ssc8: 48183518-48277476
MARC0024662	8	51261272	1.76E-08	*SNORA18*	ENSSSCG00000018686	83014	ssc8: 51344286-51344401
MARC0076384	8	51526322	1.76E-08	*SNORA18*	ENSSSCG00000018686	181921	ssc8: 51344286-51344401
H3GA0024878	8	52127966	1.76E-08	*NPY1R*	ENSSSCG00000008888	24683	ssc8: 52152649-52163080
MARC0017124	8	52401971	1.76E-08	*MARCH1*	ENSSSCG00000039175	within	ssc8: 52375510-52951031

ALGA0047893	8	52501676	1.76E-08	*MARCH1*	ENSSSCG00000039175	within	ssc8: 52375510-52951031
ASGA0038818	8	52886452	1.76E-08	*MARCH1*	ENSSSCG00000039175	within	ssc8: 52375510-52951031
MARC0036671	8	56669257	1.76E-08	*IGFBP7*	ENSSSCG00000008913	311435	ssc8: 56283089-56357822
MARC0001802	8	58272180	1.76E-08	*IGFBP7*	ENSSSCG00000008913	1914358	ssc8: 56283089-56357822
ALGA0114185	8	58525989	1.76E-08	*IGFBP7*	ENSSSCG00000008913	2168167	56283089-56357822
INRA0029837	8	59232142	1.76E-08	NA			
MARC0064305	8	59455986	1.76E-08	NA			
H3GA0024902	8	60060727	1.76E-08	*ADGRL3*	ENSSSCG00000026129	within	ssc8: 59988280-60800177
MARC0008870	8	60232132	1.76E-08	*ADGRL3*	ENSSSCG00000026129	within	ssc8: 59988280-60800177
DRGA0008598	8	60731805	1.76E-08	*ADGRL3*	ENSSSCG00000026129	within	ssc8: 59988280-60800177
DRGA0008609	8	63495578	1.76E-08	*EPHA5*	ENSSSCG00000008919	within	ssc8: 63285006-63604734
DRGA0008621	8	65528450	1.76E-08	*UBA6*	ENSSSCG00000008924	57621	ssc8: 65381058-65470829
MARC0048950	8	66655594	1.76E-08	*UGT2A3*	ENSSSCG00000034976	within	ssc8: 66646240-66663405
MARC0070956	8	66531049	1.76E-08	*UGT2A3*	ENSSSCG00000034976	115191	ssc8: 66646240-66663405
H3GA0024939	8	67647137	1.76E-08	*DCK*	ENSSSCG00000008942	59524	ssc8: 67706661-67736743
WU_10.2_8_71948204	8	68075431	1.76E-08	*SLC4A4*	ENSSSCG00000008943	within	ssc8: 67768955-68177213
WU_10.2_8_72005448	8	68132567	1.76E-08	*SLC4A4*	ENSSSCG00000008943	within	ssc8: 67768955-68177213
WU_10.2_8_148222960	8	66908768	1.76E-08	*CSN1S1*	ENSSSCG00000009262	462	ssc8: 66888231-66908306
WU_10.2_8_148366150	8	67118704	1.76E-08	*CSN3*	ENSSSCG00000009267	9592	ssc8: 67099131-67109112
WU_10.2_8_148440405	8	67104638	1.76E-08	*CSN3*	ENSSSCG00000009267	within	ssc8: 67099131-67109112
WU_10.2_8_148481247	8	67096284	1.76E-08	*CSN3*	ENSSSCG00000009267	2847	ssc8: 67099131-67109112
WU_10.2_8_148484852	8	67092680	1.76E-08	*CSN3*	ENSSSCG00000009267	6451	ssc8: 67099131-67109112
WU_10.2_8_148485522	8	67092010	1.76E-08	*CSN3*	ENSSSCG00000009267	7121	ssc8: 67099131-67109112
ALGA0047879	8	49804202	2.66E-08	*U6*	ENSSSCG00000020037	296087	ssc8: 50100289-50100395
H3GA0024944	8	70036824	3.98E-08	*PPBP*	ENSSSCG00000037579	14334	ssc8: 70021468-70022490
ALGA0047920	8	55494829	4.37E-08	*KIAA1211*	ENSSSCG00000008903	within	ssc8: 55336363-55600931
ASGA0103358	8	36902683	5.36E-08	*GABRA4*	ENSSSCG00000030515	within	ssc8: 36875649-36943016
ALGA0105374	8	37495537	5.36E-08	*ATP10D*	ENSSSCG00000008812	within	ssc8: 37407677-37531707
WU_10.2_8_39735738	8	37795537	5.36E-08	*CORIN*	ENSSSCG00000008813	within	ssc8: 37530812-37811226
DRGA0008560	8	37056024	5.36E-08	*GABRB1*	ENSSSCG00000035621	within	ssc8: 36978014-37354950
WU_10.2_8_39105768	8	37183776	5.36E-08	*GABRB1*	ENSSSCG00000035621	within	ssc8: 36978014-37354950

MARC0064308	8		5.36E-08				
ASGA0038709	8	37868900	5.36E-08	*NFXL1*	ENSSSCG00000008816	within	ssc8: 37812378-37875582
ALGA0047689	8	37881067	5.36E-08	*NFXL1*	ENSSSCG00000008816	5485	ssc8: 37812378-37875582
WU_10.2_8_39955647	8	37966306	5.36E-08	*NIPAL1*	ENSSSCG00000037950	2814	ssc8: 37936812-37963492
WU_10.2_8_40085897	8	37953470	5.36E-08	*NIPAL1*	ENSSSCG00000037950	within	ssc8: 37936812-37963492
ASGA0038720	8	38339432	5.36E-08	*SLAIN2*	ENSSSCG00000008821	18769	ssc8: 38241497-38320663
H3GA0024811	8	39096198	6.08E-08	*LRRC66*	ENSSSCG00000008832	8529	ssc8: 39104727-39127021
WU_10.2_8_40573763	8	38532503	6.08E-08	*FRYL*	ENSSSCG00000008826	within	ssc8: 38395780-38745838
ALGA0047711	8	38710937	6.08E-08	*OCIAD1*	ENSSSCG00000008828	34478	ssc8: 38745415-38769255
MARC0026493	8	36946122	7.42E-08	*GABRA4*	ENSSSCG00000030515	3106	ssc8: 36875649-36943016
ALGA0108352	8		9.16E-08				
MARC0111015	8	65241398	1.04E-07	*CENPC*	ENSSSCG00000008921	within	ssc8: 65237825-65315788
MARC0056888	8	49328663	1.04E-07	*U6*	ENSSSCG00000020037	771626	ssc8: 50100289-50100395
H3GA0054093	8	38154443	1.25E-07	*TEC*	ENSSSCG00000008820	within	ssc8: 38051053-38182259
WU_10.2_8_43364095	8	41273714	1.7E-07	*PDGFRA*	ENSSSCG00000008841	252274	ssc8: 40966568-41021440
WU_10.2_8_43641500	8	41532565	1.7E-07	*KIT*	ENSSSCG00000008842	40259	ssc8: 41402334-41492306
MARC0038980	8	48773981	1.86E-07	*RAPGEF2*	ENSSSCG00000008881	496505	ssc8: 48183518-48277476
WU_10.2_8_40595314	8	38554055	2.45E-07	*FRYL*	ENSSSCG00000008826	within	ssc8: 38395780-38745838

SNP, single nucleotide polymorphisms; [1]*Sus scrofa* chromosome; [2]Derived from the current porcine genome sequence assembly (Sscrofa11.1) (http://www.ensembl.org/Sus_scrofa/Info/Index); NA: not assigned.
